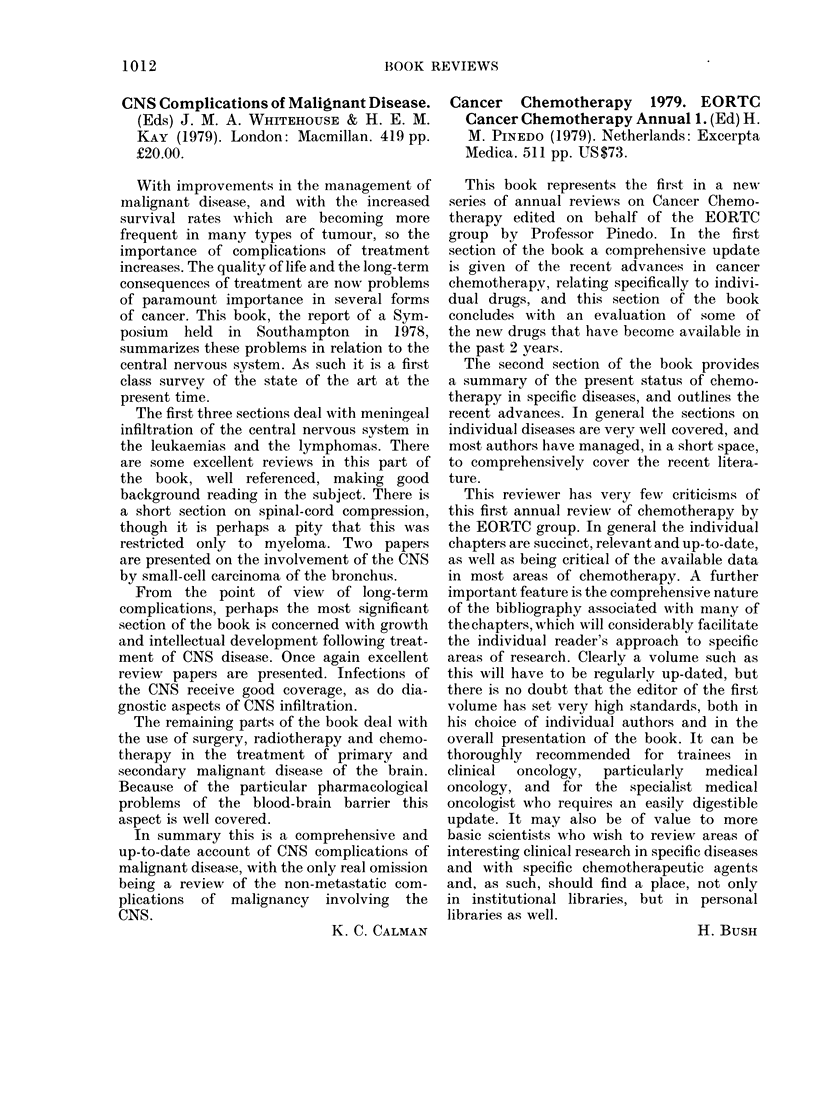# Cancer Chemotherapy 1979. EORTC Cancer Chemotherapy Annual 1

**Published:** 1980-06

**Authors:** H. Bush


**Cancer Chemotherapy 1979. EORTC Cancer Chemotherapy Annual 1.** (Ed) H. M. Pinedo (1979). Netherlands: Excerpta Medica. 511 pp. US$73.

This book represents the first in a new series of annual reviews on Cancer Chemotherapy edited on behalf of the EORTC group by Professor Pinedo. In the first section of the book a comprehensive update is given of the recent advances in cancer chemotherapy, relating specifically to individual drugs, and this section of the book concludes with an evaluation of some of the new drugs that have become available in the past 2 years.

The second section of the book provides a summary of the present status of chemotherapy in specific diseases, and outlines the recent advances. In general the sections on individual diseases are very well covered, and most authors have managed, in a short space, to comprehensively cover the recent literature.

This reviewer has very few criticisms of this first annual review of chemotherapy by the EORTC group. In general the individual chapters are succinct, relevant and up-to-date, as well as being critical of the available data in most areas of chemotherapy. A further important feature is the comprehensive nature of the bibliography associated with many of the chapters, which will considerably facilitate the individual reader's approach to specific areas of research. Clearly a volume such as this will have to be regularly up-dated, but there is no doubt that the editor of the first volume has set very high standards, both in his choice of individual authors and in the overall presentation of the book. It can be thoroughly recommended for trainees in clinical oncology, particularly medical oncology, and for the specialist medical oncologist who requires an easily digestible update. It may also be of value to more basic scientists who wish to review areas of interesting clinical research in specific diseases and with specific chemotherapeutic agents and, as such, should find a place, not only in institutional libraries, but in personal libraries as well.

H. Bush